# Having an abortion during your PhD

**DOI:** 10.7554/eLife.82643

**Published:** 2022-08-22

**Authors:** Amanda Smythers

**Affiliations:** 1 Department of Chemistry, University of North Carolina at Chapel Hill Chapel Hill United States

**Keywords:** sparks of change, research culture, equity diversity and inclusion, abortion, reproductive rights

## Abstract

A graduate student reflects on her choice to end a pregnancy, and on what the overturning of Roe v. Wade means for trainees in the United States.

It’s January 2020. I am on the brink of my 30th birthday, a first-year PhD student in chemistry at the University of North Carolina at Chapel Hill, and I am laying on a hospital cot with my legs up in stirrups. Instead of studying for my exam the following morning, I am having an abortion.

The doctor asks: “Do you want us to stop”?

I realize I have been crying involuntarily, the heaviness of the moment weighing on me more than anticipated. I tell the doctor I want to continue, and squeeze my eyes shut. That evening, I hug my daughter and fall into an uncomfortable sleep. I wake up the next day, take an exam. Life moves on.

My PhD started only six months before, the culmination of years of hard work and dedication. After having bounced around jobs, in 2014 I enrolled in a biology degree, hoping to create a career where I could support myself and my husband. Much to my chagrin, the course included chemistry – a topic that I had sworn in high school I would never study again. But my professor was incredible, and my trajectory changed forever.

Within six months, I had started conducting research in a lab, changed my degree to biochemistry, and knew I wanted to pursue a PhD. I took six weeks off that summer to welcome my daughter into the world, and then went back to work full time. Throughout her first months I wrangled coursework, research and motherhood, on top of serving as a teaching assistant and an outreach volunteer. I went on to obtain an MSc, co-author five papers, and most important, receive a PhD offer at an institution I never dreamed I would be able to attend. My goal was straightforward: I would work as hard as I could to finish my PhD in four years. I would become a productive researcher and thoughtful scientific citizen, move on to a postdoc, and eventually find a tenure-track position.

My first semester at UNC Chapel Hill was both intellectually exhilarating and exhausting as I had to sit for exams as part of my PhD. On top of this, I was put on a waiting list to receive financial help for childcare. This meant that half of my stipend went to my daughter’s creche every month, a bill that weighed heavily on the finances of my family. But the end was in sight: my daughter was less than a year away from starting kindergarten, and I would soon finish exams and advance to the research-only stage of my program.

At the end of the semester, however, I could tell something was wrong. I was consistently nauseous, often gagging when someone opened an organic solvent in the lab. I would get lightheaded if I moved too quickly and my favourite takeout made me sick to my stomach. Still, it wasn’t until a friend mentioned it that I even considered I could be pregnant. Once confirmed by my gynaecologist, I couldn’t stop crying. Unlike my first pregnancy, these were not tears of joy.

I love being a mom. My daughter provides joy in my life that I never thought was possible. But the thought of having another child terrified me. Daycare costs in Chapel Hill would cause an enormous financial burden and my family was already struggling. My graduate student health insurance does not cover spouses or dependents, and it was unclear whether my department would provide paid maternity leave. And while it made me feel selfish, I knew it would be impossible to maintain the high level of productivity needed to create the academic career I was working toward. I have love to give in excess, but it will never pay my family’s bills, make up for my socioeconomic class or sustain my career aspirations. With my husband’s support, I made the appointment for an abortion at our local Planned Parenthood. I was relieved that there were no protesters on the day of my procedure. I have no regrets.

I was in the lab when my phone chimed and I learned that Roe v. Wade had been officially overturned by the Supreme Court. For me, this was a fitting setting to receive this news. I can say with certainty that, if stripped of my right to choose, I wouldn’t still be in my doctoral program today. I wouldn’t have co-authored 10 articles over the past three years and been awarded any of the fellowships or prizes I’ve received for my doctoral performance. My ability to succeed as a scientist would have been severely compromised; my family’s economic mobility would have been stopped in its tracks.

Reversing Roe v. Wade will be detrimental to the health and opportunities of anyone who can become pregnant. It will harm children whose parents are already struggling. It will disproportionately affect those who cannot travel across state lines. It is cruel and theocratic in nature.

Some will claim that abortion rights and scientific research do not intersect, but this cannot be farther from the truth. Trainees will be among the most severely impacted by a loss of reproductive freedom, especially those from communities which are already underrepresented in science. I believe that this will be the case as long as academic culture remains so unwelcoming for parents, and in particular for mothers. I am doing the best I can with what I have available to me. Everybody, every woman, should be given that right.

Scientists should be outraged by the removal of reproductive freedom across the United States: if not for the safety and success of their trainees, then at least because it defies basic scientific truths – from equating embryonic cardiac activity with a “heartbeat” to denying the fact that legal abortions are crucial for healthcare and medically safe.

While some high-profile scientific outlets have responded forcefully in favour of reproductive justice, I have seen very little outrage – and even fewer tangible actions – from those who can influence policy, especially at research institutions. Some of these decision-makers may consider themselves ‘pro-life’. But what is life without dignity, and what is dignity if not the ability to make decisions over your own body? Why take a position of power and privilege if you don’t intend to use it – and if not now, then when?

Academic scientists, in particular, need to step up to support colleagues and trainees who may face unconscionable decisions in the near future. We need policies to protect those who will seek an abortion across state lines, including paid time off and financial compensation if insurance refuse their claim. We need policies that support those who decide to keep their pregnancies, so that no one needs to decide between feeding their current family and bringing another child into existence. We need explicit support of the freedom to choose, not just on Twitter, and not just on days surrounding major news stories. We need you now.

The removal of fundamental reproductive rights is shameful. Watching celebrations of anti-abortion advocates hurts, but the silence of our allies hurts more. To those in positions of power, I beg you: speak up and take action before it is too late.

## Share your experiences

This article is a Sparks of Change column, where people around the world share moments that illustrate how research culture is or should be changing. Have an interesting story to tell? See what we’re looking for and the best ways to get in touch here.

